# Brief Report: Reassessment of bTMB as a Predictive Biomarker in NSCLC Immunotherapy—A Reanalysis of OAK and POPLAR

**DOI:** 10.1016/j.jtocrr.2026.101066

**Published:** 2026-08-31

**Authors:** Jiaqian Liu, Na Bo, Patrick Nana-Sinkam, Ying Ding

**Affiliations:** a Department of Biostatistics and Health Data Science, School of Public Health, University of Pittsburgh, Pittsburgh, Pennsylvania; b Department of Biostatistics, School of Public Health, Virginia Commonwealth University, Richmond, Virginia; c Division of Pulmonary and Critical Care, Virginia Commonwealth University, Richmond, Virginia; d Wright Regional Center for Clinical and Translational Science, Virginia Commonwealth University, Richmond, Virginia

**Keywords:** Atezolizumab, Blood-based tumor mutational burden, Differential treatment efficacy, Non–small cell lung cancer, Predictive biomarker

## Abstract

**Introduction:**

Blood-based tumor mutational burden (bTMB) has been proposed as a potential biomarker for identifying patients with advanced NSCLC who may benefit from immunotherapy. Prior studies have primarily focused on the high-bTMB group, which may lead to an incomplete or misleading assessment of the predictive value of bTMB. In this study, we used data from the POPLAR (NCT01903993) and OAK (NCT02008227) trials to re-evaluate the predictive role of bTMB for immunotherapy response in advanced NSCLC by analyzing all patients with available bTMB measures and evaluating outcomes in both high- and low-bTMB groups.

**Methods:**

Patients with evaluable bTMB from the POPLAR and OAK trials were included. For each study, hazard ratios (HRs) for both progression-free survival (PFS) and overall survival (OS) were calculated separately for the high- and low-bTMB groups across a range of cut points.

**Results:**

A total of 211 and 583 patients with evaluable bTMB were included from the POPLAR and OAK trials, respectively. bTMB cut points from 4 to 26 (in increments of 2) were evaluated. At a cut point of 14, one of the evaluated thresholds, in POPLAR, patients with high bTMB had a PFS HR of 0.50 (95% confidence interval [CI]: 0.30–0.83) and an OS HR of 0.51 (95% CI: 0.30–0.86), whereas patients with low bTMB had a PFS HR of 1.23 (95% CI: 0.86–1.76) and an OS HR of 0.82 (95% CI: 0.55–1.21). In OAK, patients with high-bTMB had a PFS HR of 0.68 (95% CI: 0.50–0.91), and those with low bTMB had a PFS HR of 0.98 (95% CI: 0.79–1.12), with no difference between the two groups for OS (HR = 0.63, 95% CI: 0.45–0.88 versus HR = 0.63, 95% CI: 0.50–0.80).

**Conclusions:**

The predictiveness of bTMB in identifying patients with NSCLC who may benefit from immunotherapy was evident for PFS but not for OS. Observing a differential treatment efficacy trend solely within the high-bTMB group across multiple cut points was insufficient to establish predictive utility. A valid assessment requires a comprehensive evaluation of both high- and low-bTMB populations, with predictiveness established by reporting differential treatment efficacy between these two groups.

## Introduction

Lung cancer is the most common cause of cancer-related death globally, with NSCLC accounting for approximately 80% of all lung cancer diagnoses.^1^^,^^2^ Immune checkpoint inhibitors targeting the programmed death–1 and programmed death-ligand 1 pathway represent a ground-breaking advancement in the treatment of advanced NSCLC.^3^, ^4^, ^5^, ^6^ Blood-based tumor mutational burden (bTMB) has emerged as a promising, minimally invasive biomarker for developing precision care of NSCLC.

Gandara et al.^7^ analyzed progression-free survival (PFS) and overall survival (OS) in two advanced NSCLC randomized clinical trials (POPLAR and OAK) and found that bTMB is a predictive biomarker for PFS. Predictiveness was assessed by comparing hazard ratios (HRs) between atezolizumab and docetaxel in high-bTMB groups (defined as bTMB ≥ cut point) across different cut points in POPLAR and was validated in OAK. Among patients with a bTMB of 16 or higher, a greater PFS benefit with atezolizumab was found in the biomarker-evaluable population (BEP), defined as patients with baseline plasma samples that met the bTMB assay quality criteria.^7^

Several studies, including POPLAR, OAK, Blood First-Line Ready Screening Trial,^8^ Blood-First Assay Screening Trial,^9^ and MYSTIC,^10^ have evaluated bTMB as a predictive biomarker for immune checkpoint inhibitors, reporting predictive effects on PFS or OS, consistent with Gandara et al.^7^ and other TMB-related analyses, such as the CHECKpoint Pathway and nivoluMAb Clinical Trial Evaluation studies.^11^ Nevertheless, these analyses primarily evaluated HR trends within high-bTMB subgroups and are insufficient to establish predictive effects because this approach lacks the comparative context provided by the inclusion of the low-bTMB cohort, leading to an incomplete characterization of bTMB. Predictiveness should instead be evaluated by reporting differential treatment effects between high- and low-bTMB subgroups rather than by examining efficacy trends across different cut points only among patients with bTMB greater than each cutoff. Accordingly, we re-analyzed POPLAR and OAK and reported that evaluating treatment efficacy in both high- and low-bTMB cohorts provides a more accurate characterization of bTMB’s predictiveness as a clinical biomarker for immunotherapy.

## Materials and Methods

### Datasets

Quantification of bTMB was done from plasma circulating cell-free DNA using a FoundationOne CDx–based hybrid-capture next-generation sequencing assay targeting approximately 1.1 Mb of coding genomic regions. In this study, bTMB was defined as the number of somatic, non-synonymous mutations per megabase. Measurements of bTMB were obtained from Gandara et al.^7^ In POPLAR, 211 of the total 287 patients had evaluable bTMB measurements (n = 105 on atezolizumab versus n = 106 on docetaxel). In OAK, 583 of the total 850 patients had evaluable bTMB measurements (n = 293 on atezolizumab versus n = 290 on docetaxel). These patients constituted the BEP for each study and served as the population for our analysis. The clinical endpoints considered in both trials were PFS and OS. More detailed descriptions of the original trial designs, data collection, statistical analyses, and results can be found in Fehrenbacher et al.^5^ and Rittmeyer et al.^6^ This study was a re-analysis of de-identified trial data, and additional informed consent was not required.

### Prognostic and Predictive Biomarkers

According to the Food and Drug Administration–National Institute for Health Research Biomarker Working Group,^12^ a prognostic biomarker is defined as one that is associated with differences in clinical outcomes between biomarker groups, reflecting variation in underlying disease risk. A predictive biomarker is defined as one that identifies differential treatment effects across biomarker groups. Thus, a prognostic biomarker provides information about disease outcome regardless of treatment, whereas a predictive biomarker identifies differences in treatment benefit according to biomarker status.

### Statistical Analysis

We analyzed OS and PFS for both trials. We used the same set of bTMB cut points as in Gandara et al.,^7^ ranging from 4 to 26 in increments of 2. For each cut point, patients were dichotomized into high (bTMB ≥ cut point; g^+^) and low (bTMB < cut point; g^-^) groups, and HRs for atezolizumab versus docetaxel in the g^+^ and g^-^ groups were estimated using a Cox proportional hazards model that included treatment (atezolizumab versus docetaxel), bTMB group (g^+^ versus g^-^), and their interaction. Overall HRs were estimated using a Cox model that included treatment as the only covariate in the BEP. Kaplan-Meier (KM) curves were generated to visualize the survival distributions in each treatment-by-bTMB group. All statistical analyses were performed using R version 4.3.1 (R Foundation for Statistical Computing, Vienna, Austria).

## Results

Patient characteristics were presented in Supplementary Table 1.

### POPLAR

In the POPLAR BEP, the overall HR for OS was 0.68 (95% confidence interval [CI]: 0.50–0.93). Across all cut points, OS HRs in both the g^+^ and g^-^ groups remained below 1. For example, at cut point 14, which provided clear separation between groups and simultaneously maintained reasonable subgroup sizes, 73 patients (34.6%) were classified as g^+^ and 138 patients (65.4%) as g^-^ (Fig. 1*A*). The corresponding OS HRs were 0.51 (95% CI: 0.30–0.86) for g^+^ and 0.82 (95% CI: 0.55–1.21) for g^-^. For PFS, the overall HR was 0.90 (95% CI: 0.68–1.20). In contrast to OS HRs, PFS HRs for the g^+^ group remained consistently below 1 across all cut points, whereas those for the g^-^ group were near or above 1 (Fig. 1*B*). At cut point 14, the PFS HRs were 0.50 (95% CI: 0.30–0.80) for g^+^ and 1.23 (95% CI: 0.86–1.76) for g^-^. The KM curves at this cut point (Fig. 2*A* and *B*) were consistent with these findings: for OS, greater separation between treatment arms was observed in the g^+^ group than in the g^-^ group throughout follow-up, with atezolizumab-treated patients in g^+^ having the most favorable OS. A similar pattern was observed for PFS, for which little separation between treatment arms was evident in the g^-^ group.

**Figure 1:**
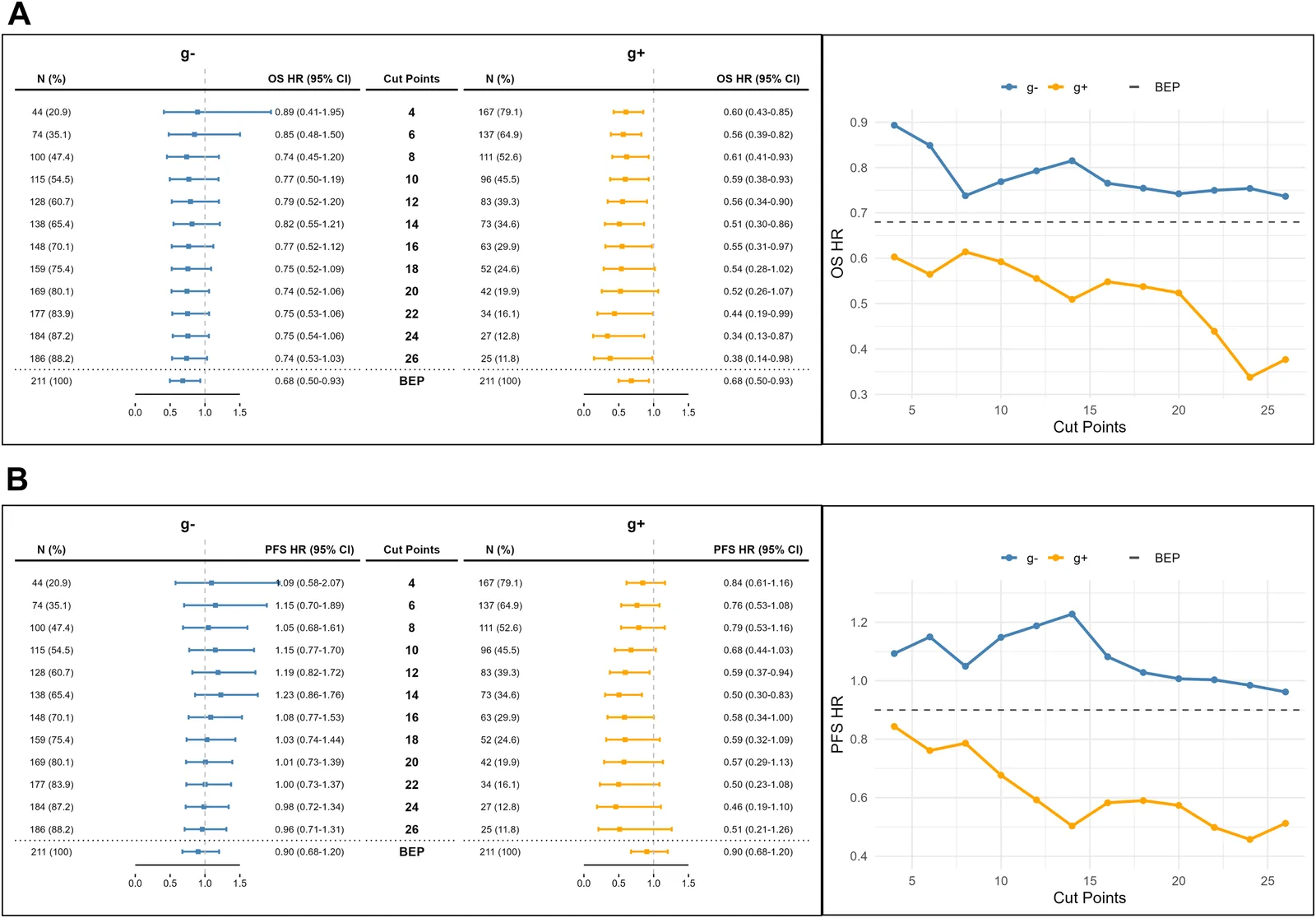
Forest plots of HRs for OS *(A)* and PFS *(B)* in g^+^ and g^-^ patients in POPLAR. N refers to the number of patients with bTMB above each cut point and the proportion of patients (%) in BEP. The error bars indicate 95% CIs. BEP, biomarker-evaluable population; bTMB, blood-based tumor mutational burden; CI, confidence interval; g^+^, bTMB higher than cut point; g^-^, bTMB lower than cut point; HR, hazard ratio; OS, overall survival; PFS, progression-free survival.

**Figure 2:**
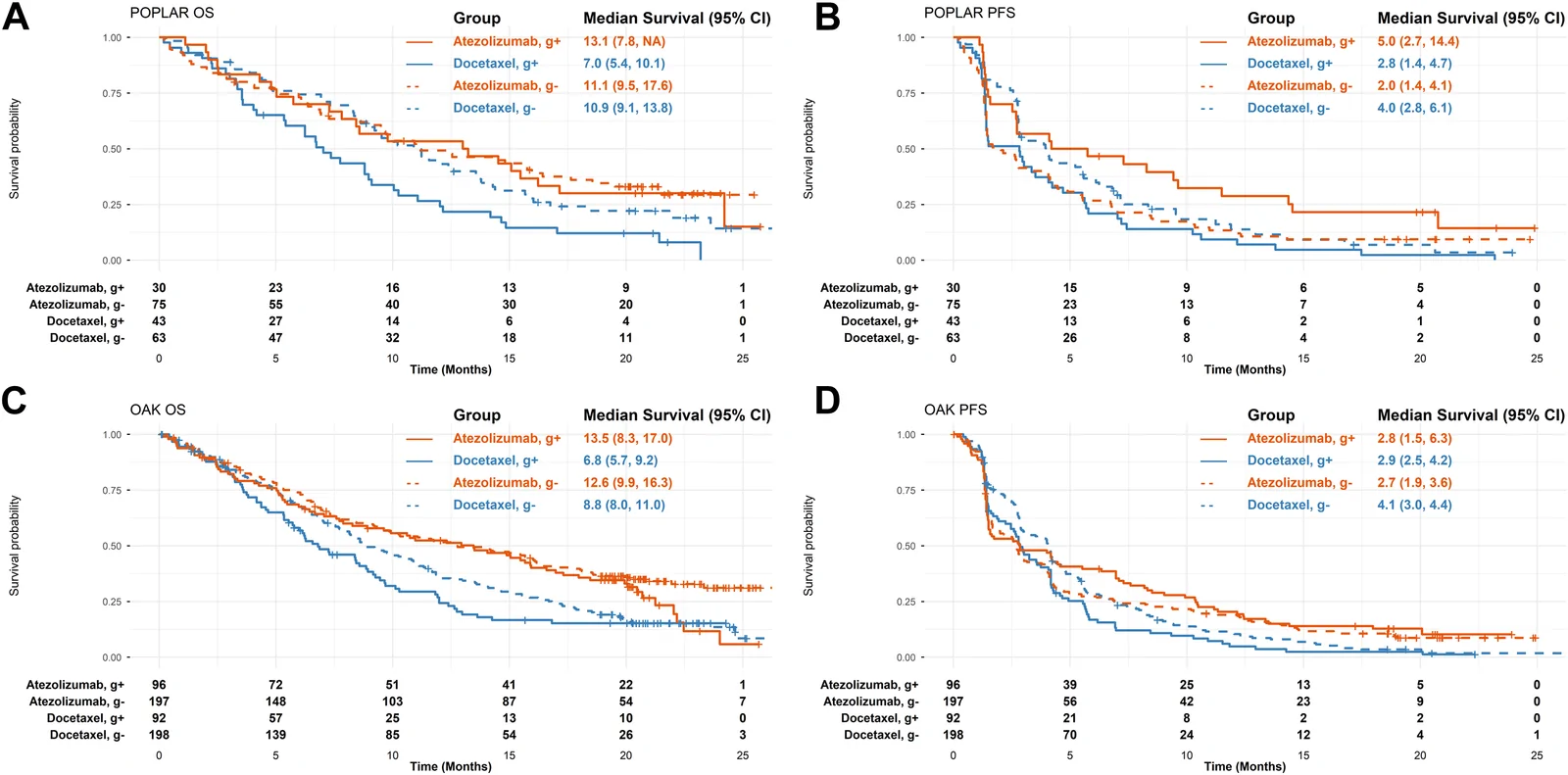
Kaplan-Meier curves for OS and PFS in POPLAR and OAK, by bTMB group (g^+^, bTMB ≥ 14; g^-^, bTMB < 14) and treatment arm. bTMB, blood-based tumor mutational burden; CI, confidence interval; g^+^, bTMB higher than cut point; g^-^, bTMB lower than cut point; OS, overall survival; PFS, progression-free survival.

### OAK

In the OAK BEP, the overall OS HR was 0.64 (95% CI: 0.53–0.77). Although HRs in g^+^ and g^-^ groups were all less than 1 across all cut points, they did not exhibit consistent separation (Fig. 3*A*). Notably, at all cut points below 14, the HR in the g^-^ group was lower than that in the g^+^ group, indicating greater treatment benefit in the g^-^ group, which contradicts the expected biological rationale for bTMB (i.e., higher tumor burden predicting greater response to immunotherapy). Above this threshold, the HRs fluctuated, with no consistent trend. At the cut point 14, 188 patients (32.2%) were classified as g^+^ and 395 patients (67.8%) as g^-^, with the same HR of 0.63 in both subgroups and similar 95% CIs (g^+^: 0.45–0.88; g^-^: 0.50–0.80). The KM curves at this cut point further supported these findings: among patients receiving atezolizumab, the g^+^ and g^-^ curves largely overlapped, indicating similar survival. In contrast, among patients receiving docetaxel, those with g^+^ exhibited poorer survival than those with g^-^, suggesting that bTMB may have a prognostic effect in patients treated with docetaxel rather than serving as a predictive biomarker for atezolizumab (Fig. 2*C*).

**Figure 3:**
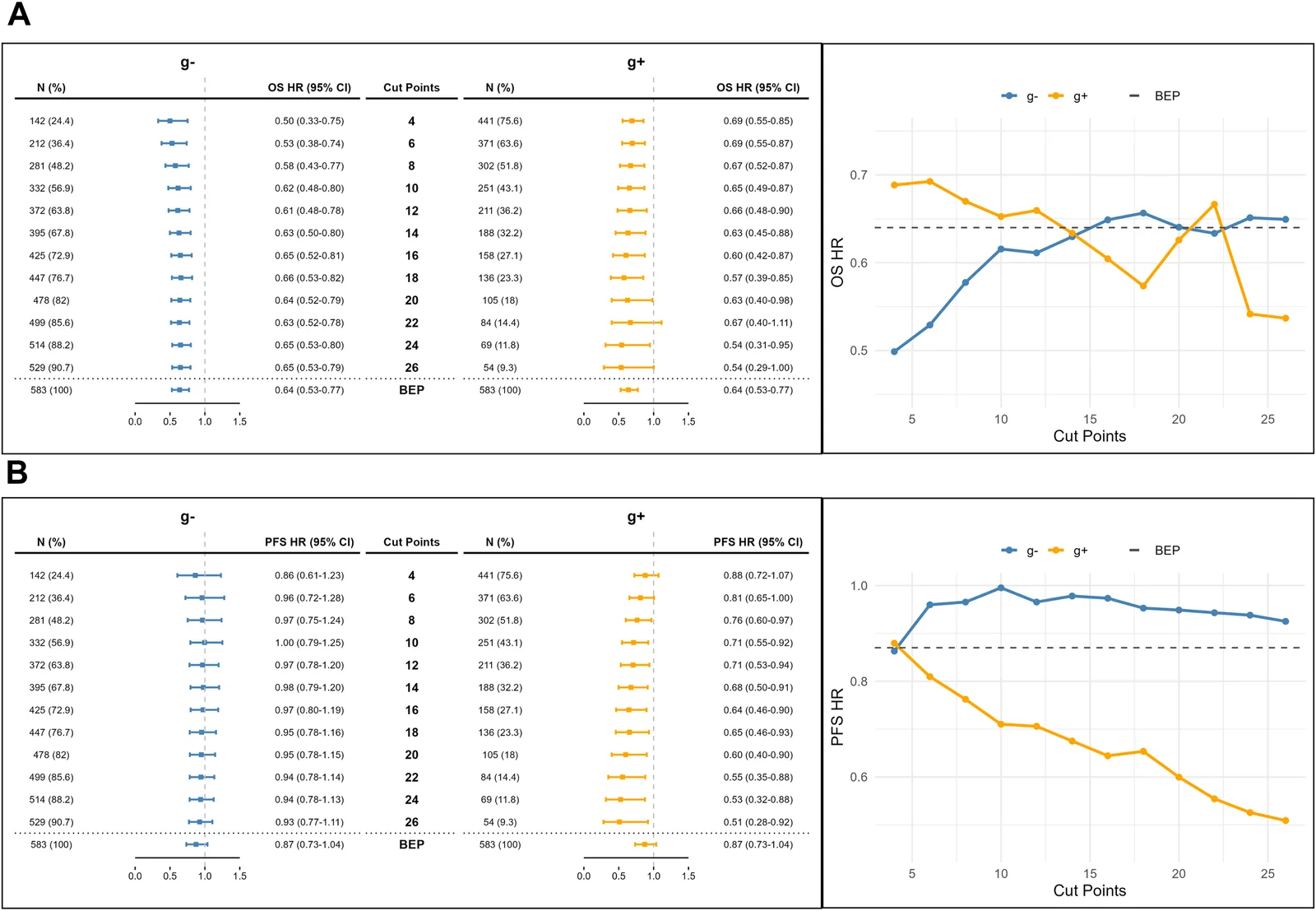
Forest plots of HRs for OS *(A) and PFS (B)* in g^+^ and g^-^ patients in OAK. N refers to the number of patients with bTMB above each cut point and the proportion of patients (%) in BEP. The error bars indicate 95% CIs. BEP, biomarker-evaluable population; bTMB, blood-based tumor mutational burden; CI, confidence interval; g^+^, bTMB higher than cut point; g^-^, bTMB lower than cut point; HR, hazard ratio; OS, overall survival; PFS, progression-free survival.

For PFS, the overall HR was 0.87 (95% CI: 0.73–1.04). The HRs in both g^+^ and g^-^ groups were less than 1, differing from what was observed in POPLAR (Fig. 3*B*). In addition, HRs for PFS in g^+^ were lower than in g^-^ across the bTMB cut points of over 2, indicating consistent separation between the two groups over almost all cut points. At the cut point of 14, the separation was pronounced; the HR was 0.68 (95% CI: 0.50–0.91) in the g^+^ group and 0.98 (95% CI: 0.79–1.20) in the g^-^ group. Although the four KM curves reported little separation early in follow-up, greater between-arm separation in g^+^ than in g^-^ became apparent after approximately 4 to 5 months (Fig. 2*D*).

## Discussion

In this study, we analyzed the patient survival benefit of atezolizumab versus docetaxel in the POPLAR and OAK trials by evaluating differential treatment effects in bTMB-defined subgroups. In summary, bTMB was predictive of PFS in both studies but remained inconclusive for OS. Specifically, HRs for OS in low-bTMB groups were less than those of high-bTMB groups at most cut points in OAK, which is inconsistent with findings from POPLAR and with biological evidence suggesting that a higher tumor mutational burden is associated with greater benefit from immunotherapy.^13^, ^14^, ^15^ The discordant OS findings between POPLAR and OAK may be related to differences in patient populations between the two studies (Supplementary Table 2). For example, compared with those in POPLAR, patients in the OAK BEP were older (61.6 versus 63.9 years, *p* < 0.01) and more likely to have received no prior therapy in the locally advanced or metastatic setting (67.8% versus 76.1%, *p* = 0.02). In addition, the OAK BEP excluded patients with positive *EGFR* mutations or EML4-ALK translocations, whereas the POPLAR BEP did not. We also repeated the analyses after propensity score matching between the two trials. After matching, the OS trends in OAK became more consistent with those in POPLAR, suggesting that differences in patient populations may explain the observed heterogeneity (Supplementary Fig. 1).

For survival endpoints, trends in HRs across cut points may still be insufficient to establish a biomarker’s predictiveness, as illustrated by the unexpected OS HR patterns in OAK for high- and low-bTMB groups. To accurately establish the biomarker’s role, additional survival measures, such as KM curves and median survival, should also be evaluated because targeting the wrong patient subgroup can lead to adverse clinical consequences. Therefore, a comprehensive assessment of both high- and low-bTMB groups using multiple survival measures is crucial for accurately interpreting the clinical utility of bTMB in NSCLC. This study has several limitations. First, as a post hoc analysis, the observed predictive performance of bTMB for PFS in POPLAR and OAK should be interpreted as exploratory rather than confirmatory, particularly given the inconclusive findings for OS. Second, the evaluation of multiple bTMB cut points did not account for multiplicity. Statistical methods to address this issue are the focus of separate, ongoing methodological work. Third, despite propensity score matching, residual differences in patient characteristics may have contributed to the observed heterogeneity between the two trials.

## CRediT Authorship Contribution Statement

**Jiaqian Liu:** Conceptualization, Methodology, Formal analysis, Visualization, Writing - original draft, Writing - review & editing.

**Na Bo:** Conceptualization, Methodology, Writing - review & editing.

**Patrick Nana-Sinkam:** Conceptualization, Writing - review & editing.

**Ying Ding:** Conceptualization, Methodology, Supervision, Writing - review & editing.

## Disclosure

The authors declare no conflict of interest.

## Footnotes

Note: To access the supplementary material accompanying this article, visit the online version of the *JTO Clinical and Research Reports* at www.jtocrr.org and at https://doi.org/10.1016/j.jtocrr.2026.101066.
