# Supplementary material for: Brief Report: Reassessment of bTMB as a Predictive Biomarker in NSCLC Immunotherapy—A Reanalysis of OAK and POPLAR
Source: JTO Clin Res Rep. 2026 Aug 31;7(11):101066. doi: 10.1016/j.jtocrr.2026.101066 (PMC13675807; doi:10.1016/j.jtocrr.2026.101066)
Supplement: Supplementary file 1 [file mmc1.docx]

**Supplemental Figures**

**Supplemental Figure 1.** Forest plots of HRs for OS in g⁺ and g⁻ patients from matched POPLAR and OAK cohorts. N refers to the number of patients with bTMB above each cut point and the proportion of patients (%) in matched populations. The error bars indicate 95% CIs.

A propensity score matching analysis was performed to improve the comparability of the POPLAR and OAK cohorts. After matching without replacement, 205 patients from POPLAR were matched to 281 patients from OAK. In the matched OAK cohort, patients in the g⁺ subgroup generally exhibited better survival than those in the g⁻ subgroup across most bTMB cut points, resulting in a pattern that was more consistent with that observed in POPLAR.
