# Supplementary material for: Brief Report: Reassessment of bTMB as a Predictive Biomarker in NSCLC Immunotherapy—A Reanalysis of OAK and POPLAR
Source: JTO Clin Res Rep. 2026 Aug 31;7(11):101066. doi: 10.1016/j.jtocrr.2026.101066 (PMC13675807; doi:10.1016/j.jtocrr.2026.101066)
Supplement: Supplementary file 2 [file mmc2.docx]

**Supplemental Figures**

**Supplemental Figure 2.** Forest plots of HRs for OS and PFS in g⁺ and g⁻ patients from the POPLAR and OAK BEP. HRs were estimated using adjusted Cox PH models controlling for age, sex, race, histology, ECOG performance status, tobacco history, and the number of previous therapies. These adjustment covariates were selected based on variables that differed significantly (*p* < 0.05) between the BEP and non-BEP in either trial. N refers to the number of patients with bTMB above each cut point and the proportion of patients (%) in BEP. The error bars indicate 95% CIs.
