# Supplementary material for: Brief Report: Reassessment of bTMB as a Predictive Biomarker in NSCLC Immunotherapy—A Reanalysis of OAK and POPLAR
Source: JTO Clin Res Rep. 2026 Aug 31;7(11):101066. doi: 10.1016/j.jtocrr.2026.101066 (PMC13675807; doi:10.1016/j.jtocrr.2026.101066)
Supplement: Supplementary file 3 [file mmc3.docx]

**Supplemental Tables**

| **Supplemental Table 1. Baseline Characteristics for POPLAR and OAK BEP** | | | | | | | |
| --- | --- | --- | --- | --- | --- | --- | --- |
| **Characteristic** | **POPLAR** | | |  | **OAK** | | |
|  | **Docetaxel (n=106)** | **Atezolizumab (n=105)** | **p-value^**^** |  | **Docetaxel (n=290)** | **Atezolizumab (n=293)** | **p-value^**^** |
| **Age** |  |  | 0.595 |  |  |  | 0.762 |
| Median [Min, Max] | 62.5  [36.0, 80.0] | 61.0  [42.0, 82.0] |  |  | 64.0  [34.0, 85.0] | 64.0  [41.0, 82.0] |  |
| Mean (SD) | 62.0 (9.1) | 61.3 (9.4) |  |  | 63.8 (9.1) | 64.0 (8.7) |  |
| **Race** |  |  | 0.102 |  |  |  | 0.914 |
| White | 89 (84.0%) | 78 (74.3%) |  |  | 213 (73.4%) | 212 (72.4%) |  |
| Asian | 8 (7.5%) | 18 (17.1%) |  |  | 54 (18.6%) | 55 (18.8%) |  |
| Other | 9 (8.5%) | 9 (8.6%) |  |  | 23 (7.9%) | 26 (8.9%) |  |
| **Sex** |  |  | 0.054 |  |  |  | 0.541 |
| Female | 48 (45.3%) | 33 (31.4%) |  |  | 107 (36.9%) | 100 (34.1%) |  |
| Male | 58 (54.7%) | 72 (68.6%) |  |  | 183 (63.1%) | 193 (65.9%) |  |
| **Histology** |  |  | >0.999 |  |  |  | 0.863 |
| Non-Squamous | 68 (64.2%) | 67 (63.8%) |  |  | 201 (69.3%) | 206 (70.3%) |  |
| Squamous | 38 (35.8%) | 38 (36.2%) |  |  | 89 (30.7%) | 87 (29.7%) |  |
| **ECOG performance status^*^** |  |  | >0.999 |  |  |  | >0.999 |
| No limitations, fully active | 30 (28.3%) | 29 (27.6%) |  |  | 99 (34.1%) | 101 (34.5%) |  |
| Limited for heavy work/strenuous activity, but able to do light, sedentary work | 76 (71.7%) | 76 (72.4%) |  |  | 191 (65.9%) | 192 (65.5%) |  |
| **Number of previous therapies in the locally advanced or metastatic setting** |  |  | 0.281 |  |  |  | 0.792 |
| 1 | 76 (71.7%) | 67 (63.8%) |  |  | 219 (75.5%) | 225 (76.8%) |  |
| 2 | 30 (28.3%) | 38 (36.2%) |  |  | 71 (24.5%) | 68 (23.2%) |  |
| **Tobacco use history** |  |  | 0.930 |  |  |  | 0.263 |
| Never | 18 (17.0%) | 17 (16.2%) |  |  | 30 (10.3%) | 42 (14.3%) |  |
| Current | 21 (19.8%) | 23 (21.9%) |  |  | 51 (17.6%) | 43 (14.7%) |  |
| Previous | 67 (63.2%) | 65 (61.9%) |  |  | 209 (72.1%) | 208 (71.0%) |  |
| **EGFR mutation** |  |  | 0.761 |  |  |  | 0.558 |
| Positive | 5 (4.7%) | 7 (6.7%) |  |  | 0 (0.0%) | 0 (0.0%) |  |
| Negative | 56 (52.8%) | 53 (50.5%) |  |  | 231 (79.7%) | 240 (81.9%) |  |
| Unknown | 45 (42.5%) | 45 (42.9%) |  |  | 59 (20.3%) | 53 (18.1%) |  |
| **EML4-ALK translocation** |  |  | 0.186 |  |  |  | 0.229 |
| Positive | 3 (2.8%) | 0 (0.0%) |  |  | 0 (0.0%) | 0 (0.0%) |  |
| Negative | 40 (37.7%) | 45 (42.9%) |  |  | 136 (46.9%) | 153 (52.2%) |  |
| Unknown | 63 (59.4%) | 60 (57.1%) |  |  | 154 (53.1%) | 140 (47.8%) |  |
| **KRAS mutation** |  |  | 0.237 |  |  |  | 0.875 |
| Positive | 10 (9.4%) | 9 (8.6%) |  |  | 23 (7.9%) | 21 (7.2%) |  |
| Negative | 13 (12.3%) | 22 (21.0%) |  |  | 63 (21.7%) | 68 (23.2%) |  |
| Unknown | 83 (78.3%) | 74 (70.5%) |  |  | 204 (70.3%) | 204 (69.6%) |  |
| *: ECOG=Eastern Cooperative Oncology Group.  **: Two sample t-tests were performed for continuous variables and Chi-squared tests were performed for categorical variables. | | | | | | | |
