# Supplementary material for: Brief Report: Reassessment of bTMB as a Predictive Biomarker in NSCLC Immunotherapy—A Reanalysis of OAK and POPLAR
Source: JTO Clin Res Rep. 2026 Aug 31;7(11):101066. doi: 10.1016/j.jtocrr.2026.101066 (PMC13675807; doi:10.1016/j.jtocrr.2026.101066)
Supplement: Supplementary file 4 [file mmc4.docx]

| **Characteristic** | **POPLAR_BEP**  N = 211 | **OAK_BEP**  N = 583 | **p-value*** |
| --- | --- | --- | --- |
| **Age** |  |  | 0.002 |
| Median [Min - Max] | 62.0 [36.0 - 82.0] | 64.0 [34.0 - 85.0] |  |
| Mean (SD) | 61.6 (9.2) | 63.9 (8.9) |  |
| **Race** |  |  | 0.105 |
| White | 167 (79.1%) | 425 (72.9%) |  |
| Asian | 26 (12.3%) | 109 (18.7%) |  |
| Other | 18 (8.5%) | 49 (8.4%) |  |
| **Sex** |  |  | 0.508 |
| Female | 81 (38.4%) | 207 (35.5%) |  |
| Male | 130 (61.6%) | 376 (64.5%) |  |
| **Histology** |  |  | 0.141 |
| Non-Squamous | 135 (64.0%) | 407 (69.8%) |  |
| Squamous | 76 (36.0%) | 176 (30.2%) |  |
| **ECOG performance status** |  |  | 0.110 |
| No limitations, fully active | 59 (28.0%) | 200 (34.3%) |  |
| Limited for heavy work/strenuous activity, but able to do light, sedentary work | 152 (72.0%) | 383 (65.7%) |  |
| **Number of previous therapies in the locally advanced or metastatic setting** |  |  | 0.022 |
| 0 | 143 (67.8%) | 444 (76.2%) |  |
| 1 | 68 (32.2%) | 139 (23.8%) |  |
| **Tobacco use history** |  |  | 0.053 |
| Previous | 132 (62.6%) | 417 (71.5%) |  |
| Current | 44 (20.9%) | 94 (16.1%) |  |
| Never | 35 (16.6%) | 72 (12.3%) |  |
| **EGFR mutation** |  |  | <0.001 |
| Positive | 12 (9.9%) | 0 (0.0%) |  |
| Negative | 109 (90.1%) | 471 (100.0%) |  |
| Unknown | 90 | 112 |  |
| **EML4-AL translocation** |  |  | 0.014 |
| Positive | 3 (3.4%) | 0 (0.0%) |  |
| Negative | 85 (96.6%) | 289 (100.0%) |  |
| Unknown | 123 | 294 |  |
| **KRAS mutation** |  |  | 0.204 |
| Positive | 19 (35.2%) | 44 (25.1%) |  |
| Negative | 35 (64.8%) | 131 (74.9%) |  |
| Unknown | 157 | 408 |  |
| *Welch Two Sample t-test; Pearson's Chi-squared test | | | |
|  | | | |
